# The application of human reliability analysis to carpal tunnel decompression

**DOI:** 10.1308/rcsann.2023.0038

**Published:** 2023-06-29

**Authors:** Á Lucey, S Kennedy, A Hussey, N McInerney, JL Kelly, KM Joyce

**Affiliations:** Galway University Hospital, Ireland

**Keywords:** Carpal tunnel decompression, Human reliability analysis, Hierarchal task analysis, Human error identification

## Abstract

**Introduction:**

Many surgical procedures are prone to human error, particularly in the learning phase of skills acquisition. Task standardisation has been suggested as an approach to reducing errors, but it fails to account for the human factors associated with learning. Human reliability analysis (HRA) is a structured approach to assess human error during surgery. This study used HRA methodologies to examine skills acquisition associated with carpal tunnel decompression.

**Methods:**

The individual steps or subtasks required to complete a carpal tunnel decompression were identified using hierarchical task analysis (HTA). The systematic human error reduction and prediction approach (SHERPA) was carried out by consensus of subject matter experts. This identified the potential human errors at each subgoal, the level of risk associated with each task and how these potential errors could be prevented.

**Results:**

Carpal tunnel decompression was broken down into 46 subtasks, of which 21 (45%) were medium risk and 25 (55%) were low risk. Of the 46 subtasks, 4 (9%) were assigned high probability and 18 (39%) were assigned medium probability. High probability errors (>1/50 cases) included selecting incorrect tourniquet size, failure to infiltrate local anaesthetic in a proximal-to-distal direction and completion of the World Health Organization (WHO) surgical sign-out. Three (6%) of the subtasks were assigned high criticality, which included failure to aspirate before anaesthetic injection, whereas 21 (45%) were assigned medium criticality. Remedial strategies for each potential error were devised.

**Conclusions:**

The use of HRA techniques provides surgeons with a platform to identify critical steps that are prone to error. This approach may improve surgical training and enhance patient safety.

## Introduction

Carpal tunnel decompression, indicated for median nerve entrapment at the wrist, is one of the most common elective hand procedures, with approximately 1,700 being performed per year in Ireland with an incidence of 3.54 per 10,000.^1^ Although complications rates are typically low (reported incidence range 0.07%–1.2%), the procedure can result in unsatisfactory patient outcome.^2–4^ Carpal tunnel decompression is one of the first procedures a trainee surgeon will learn to perform independently given the numbers performed and the perceived low complexity. However, the early phase of learning may be prone to human error.^5^ Standardisation has been suggested as an approach to reducing error during learning but does not account for the impact of any individual step in the process.^6^

Human reliability analysis (HRA) is an approach that identifies systematically the impact of human error on a system to reduce adverse events and complications.^7^ First developed for use in aviation, HRA has been adopted widely by many industries, including the military and nuclear power generation.^8,9^ Recent applications of HRA in medicine include adoption by anesthesia, radiology and laparoscopic surgery.^6,10–13^ This study is the first to apply HRA in hand surgery.

Hierarchal task analysis (HTA) and the systematic human error reduction and prediction approach (SHERPA) are two recognised techniques in HRA. HTA refers to the systematic decomposition of a procedure into its component steps with a specific focus on the human factors that contribute to a safe outcome.^10^ Analyses are dependent on multiple subjective observations and recording of variations in expert clinical practice to produce a single accepted optimum method for successful task completion.^10^ SHERPA is an error reduction and prediction approach. SHERPA identifies credible errors in a process and suggests methods of error mitigation at each step. HTA serves as a framework for the application of SHERPA to identify the errors.

Applying HRA techniques such as HTA and SHERPA to carpal tunnel decompression allows trainees to view the steps in a concise manner, showing sequential tasks required to complete the procedure, highlights steps prone to error and alerts the trainee to steps in which error criticality is high.

This study aims to develop an HTA for carpal tunnel decompression and to analyse identified errors using SHERPA methodology. The resultant framework serves as a useful tool for surgical trainers and trainees.

## Methods

A standard approach for completing an HTA and SHERPA was adopted.^6^ This approach consisted of three stages: firstly, the identification of the procedure for analysis, followed by the HTA to identify key subtasks and finally the SHERPA analysis. Each of these three stages are outlined below.

### Identification of procedures for analysis

Carpal tunnel decompression typically follows a well-recognised sequence of surgical steps, taking into account anatomical variation. If an error occurs at a critical step, the consequences can lead to permanent disability for the patient. These factors mean that carpal tunnel decompression is eminently suitable for HTA.

### Hierarchal task analysis

HTA enables the systematic distillation of a carpal tunnel decompression procedure into its component steps. These subtasks are chosen with a specific emphasis on the human errors that can occur. We produced an HTA that identified all errors that could likely occur and the mitigating avoidance steps needed to prevent them.

#### Literature review:

A detailed literature review was conducted to identify relevant literature relating to the appropriate technique and best practice for carpal tunnel decompression. An extensive search of PubMed, Medline, UpToDate and Scopus was performed to construct an initial HTA for carpal tunnel decompression.^14,15^ Notable findings include the risk of iatrogenic median nerve injury, reported in 0.55% cases.^16^ Up to 70% of iatrogenic median nerve injuries occur during carpal tunnel decompression.^17^ Postoperative surgical site infection is a rare but serious complication following open carpal tunnel decompression. The 30-day infection rates after surgery procedure are between 0.32% and 0.65% when performed in a primary care setting.^18–20^ The American Society for Surgery of the Hand (ASSH) estimate the incidence of wrong site surgery in hand surgery as 1 in 27,686 or 0.00003%.^21,22^

#### Observation:

A total of 20 observations of the carpal tunnel decompression procedure were performed in our institution between January and July 2022. Four consultant plastic surgeons (Tang and Giddins Grade III–IV), performed all cases.^23^ During each observation, the steps taken by the experts were recorded and used to modify the task list.

#### Subject matter experts:

Five consultant plastic surgeons were then recruited as subject matter experts (SMEs) for the development of the final HTA. The SMEs performed the initial review of the data gathered from the literature and procedure observations. This iterative process refined the HTA until a single, optimum method for the completion of the task was constructed.

### Construction of HTA

The initial HTA was constructed by an experienced plastic surgery fellow and a consultant plastic surgeon. A standard approach was used to develop this, as described below.

• The overall goal is identified (e.g. completion of a carpal tunnel decompression)
• The series of steps that are required to achieve this goal are identified; these are known as subtasks. It is a matter of judgement as to the level of detail assigned to individual subtasks. To illustrate, a subgoal could be “complete WHO Time Out Checklist”, or it could be more detailed (i.e. confirm all team members have identified themselves by name and role, verbally confirm the patient, site and procedure, has antibiotic prophylaxis been given, has essential imaging been displayed, etc). The level of detail required was determined by consensus between the five SMEs carrying out the SHERPA based upon whether further decomposition was deemed to add little value.

### Systematic human error reduction and prediction approach

Based on literature review and expert consensus, the probability of each error was assigned along with its criticality. Once the error was identified, an error mitigation strategy was suggested. The SHERPA analyses were carried out by the same five surgeons who constructed the HTAs (Tang and Giddins Grade III, IV).^23^ The five surgeons worked together to carry out the SHERPA analysis. Any disagreements were resolved through discussion until consensus was reached. The method used to complete the SHERPA analysis was as follows:

• subtasks were classified based on the behaviour involved, from the following:
  o action (e.g. incise skin along full skin marking),
  o retrieval (e.g. complete WHO sign-out checklist),
  o checking (e.g. checking LA has provided adequate anaesthesia),
  o selection (e.g. choosing correct size tourniquet for patient),
  o information communication (e.g. complete WHO time-out checklist).
• Using the classification of error types (Table 1), errors were determined that could credibly occur during performance of the different subtasks.
• The consequences of each identified potential error were described. Probability was based on literature review and expert consensus. The probability of an error was assigned to one of the following four levels:
  (1)  low, <1/1,000
  (2)  medium, >1/1,000 but <1/100
  (3)  high, >1/100 but <1/50; and
  (4)  very high, >1/50.^6,24^
• The “recovery potential” of each error was described. For example, points occurring later in the HTA where the error could be identified before it had an effect were noted.
• The “criticality” of each error was rated using three levels:
  o low, unnoticeable clinical effect
  o medium, transient clinical effect
  o high, permanent clinical effect.^6,24^
• The probability and criticality scores were multiplied together to calculate the level of risk. A score from 0 to 2 was considered “low risk”, from 3 to 6 “medium-risk” and 7 to 12 “high-risk”.
• Potential remedial strategies were suggested to prevent each error from occurring or propagating at the individual level, the equipment level, the environmental level and the organisational level.

**Table 1:** Error classification used in SHERPA

ERROR TYPE	ERROR MODE
ACTION	A1 Too long or too short
	A2 Mistimed
	A3 Wrong direction
	A4 Too little/too much
	A5 Misaligned
	A6 Wrong object
	A7 Wrong action
	A8 Omitted
	A9 Incomplete
	A10 Wrong action on wrong object
RETRIEVAL	R1 Information not obtained
	R2 Wrong information obtained
	R3 Information retrieval incomplete
CHECKING	C1 Omitted
	C2 Incomplete
	C3 Wrong object
	C4 Wrong check
	C5 Mistimed
	C6 Wrong check, wrong object
SELECTION	S1 Omitted
	S2 Wrong selection made
INFORMATION COMMUNICATION	I1 Information not communicated
	I2 Wrong information communicated
	I3 Information communication incomplete

## Results

A total of 20 open carpal tunnel decompression procedures, performed by four consultant plastic surgeons, were observed for the purposes of the study. These observations were then used to further refine the HTA that had been constructed already through literature review and experience from two of the SMEs (Tang and Giddins Grade III–IV).^23^ A simplified carpal tunnel HTA is demonstrated in Table 2. The complete HTA, with all subtasks described, is also shown (Supplemental Material A); 13 principal tasks and 46 subtasks were identified.

**Table 2:** Simplified carpal tunnel decompression HTA

Identifier	Task
	Carpal tunnel decompression
1	WHO sign in
2	Patient preparation and tourniquet fitting
3	Planning incision
3.1	Identify Kaplans cardinal line
3.2	Identify radial border ring finger
3.3	Identify distal wrist crease
3.4	Draw the incision lines
4	LA administration
4.1	Prepare syringes with LA
4.2	Remove air from syringe and apply appropriate size infiltration needle
4.3	Swab infiltration area
4.4	Puncture the skin
4.5	Aspirate the syringe before infiltrating tissue
4.6	Infiltrate LA starting proximally and ending distally, ensuring LA infiltration is proximal enough
4.7	Safely withdraw needle and discard sharps
5	Surgical field preparation
6	WHO time-out
7	Tourniquet inflation
8	Incision
8.1	Check LA has provided appropriate anaesthesia
8.2	Position the hand to full supination
8.3	Incise skin along full marking
8.4	Deepen the incision
9	Dissection and operation
9.1	Dissection into fat
9.2	Insert retractors, i.e. cats paws or self-retainer
9.3	Division of palmar fascia
9.4	Retraction of soft tissue
9.5	Diathermy to Palmaris Brevis if present
9.6	Cutting through TCL centrally to identify underlying median nerve
9.7	Release of TCL distally to level of fat pad
9.8	Reposition retractors proximally
9.9	Clear above and below TCL proximally using Metz scissors with tips curving into ulnar direction
9.10	Release of TCL proximal to level of forearm fascia
9.1	Inspection of median nerve
10	Release tourniquet and haemostasis
10.1	Release tourniquet
10.2	Diathermy to achieve haemostasis
11	Skin closure
12	Postoperative wound care
12.1	Wet wash and dry of wound
12.2	Application of nonadherent dressing, gauze, wool, cling and crepe bandage
12.3	Remove the drapes and the tourniquet
13	WHO sign-out

Table 3 demonstrates the SHERPA analysis for carpal tunnel decompression. Of the errors identified at each subtask, the maximum risk score assigned was 6, which falls into the medium-risk category. A total of four subtasks had a risk score of 6, with a total of 18 subtasks meeting the criteria for the medium risk category. This highlights which errors have the potential for permanent clinical effect. High probability errors (>1/100 and <1/50 cases) included selecting incorrect tourniquet size, failure to sterilise the skin before administration of local anaesthetic (LA), failure to infiltrate LA in a proximal-to-distal direction and failure to complete the WHO surgical sign-out.

**Table 3:** SHERPA analysis for carpal tunnel decompression

	Error	Probability	Criticality	Risk score	Remediation
1	WHO sign-in				
1.1	WHO sign-in checklist not completed	Medium [2]	Medium [2]	4	Have checklist readily available in theatre
2	Patient preparation and tourniquet fitting				
2.1	Incorrect arm positioned and exposed	Low [1]	Low [1]	1	Ensure correct laterality of arm being positioned and exposed
2.2	Wool not applied before tourniquet application	Low [1]	Medium [2]	2	Remember to apply wool before tourniquet application
2.3	Overly large or small tourniquet selected	High [3]	Low [1]	3	Ensure a wide variety of tourniquet sizes is available in theatre
2.4	Tourniquet applied upside down	Low [1]	Low [1]	1	Ensure inflation tube is directed proximally
2.5	Theatre lights not directed to operating field	Low [1]	Low [1]	1	Remember to reposition the theatre lights
3	Planning incision				
3.1	Kaplans line misidentified	Medium [2]	Medium [2]	4	Widespread teaching on identifying anatomical landmarks
3.2	Radial border of ring finger misidentified	Medium [2]	Medium [2]	4	Widespread teaching on identifying anatomical landmarks
3.3	Distal wrist crease misidentified	Medium [2]	Medium [2]	4	Widespread teaching on identifying anatomical landmarks
3.4	Incision lines drawn at wrong anatomical landmarks	Medium [2]	Medium [2]	4	Widespread teaching on identifying anatomical landmarks
4	LA administration				
4.1	Incorrect LA drawn up	Low [1]	Low [1]	1	Ensure LA is double checked by a colleague
4.2	Air is expelled before local anaesthetic administration	Low [1]	High [3]	3	Expel air before local anaesthetic administration
4.3	Infiltration area not sterilised	High [3]	Medium [2]	6	Availability of alcohol swabs in theatre
4.4	LA puncture site too deep	Low [1]	Low [1]	1	Standardised teaching on LA administration
4.5	Syringe not aspirated before infiltrating tissue	Medium [2]	High [3]	6	Ensure no blood is visible in syringe once it is aspirated
4.6	LA not infiltrated proximal enough	High [3]	Medium [2]	6	Remember to infiltrate proximally first
4.7	Sharps improperly disposed of	Low [1]	Low [1]	1	Encourage a culture of safe sharps disposal in the workplace
5	Surgical field preparation				
5.1	Poor scrub technique	Low [1]	Low [1]	1	Encourage a culture of proper surgical scrubbing in the workplace
5.2	Drapes applied before skin is cleaned	Low [1]	Medium [2]	2	Ensure the arm is cleaned to the level of the elbow before draping
5.3	Diathermy not handed off for connection	Low [1]	Low [1]	1	Ensure equipment is ready before knife-to-skin
5.4	Sterile light handles not applied	Low [1]	Low [1]	1	Ensure light handles are available on surgical trolley
6	WHO time-out				
6.1	WHO time-out checklist not completed	Medium [2]	High [3]	6	Have checklist readily available in theatre
7	Tourniquet inflation				
7.1	Arm is not exsanguinated	Low [1]	Low [1]	1	Ensure arm is elevated before exsanguination.
7.2	Tourniquet not inflated to correct pressure	Low [1]	Medium [2]	2	Encourage culture of closed loop communication in the workplace
8	Incision				
8.1	Full extent of incision not checked	Medium [2]	Medium [2]	4	Check with patient incision site is adequately anaesthetised before first cut
8.2	Hand not correctly positioned	Medium [2]	Low [1]	2	Ensure arm is adequately supinated
8.3	Skin incisional misaligned	Low [1]	Low [1]	1	Ensure area is marked before cutting
8.4	Incision extended too deep into tissue	Low [1]	Low [1]	1	Deepen the incision in a slow and controlled manner
9	Dissection and operation				
9.1	Dissection into fat is too deep	Low [1]	Low [1]	1	Deepen the incision in a slow and controlled manner
9.2	Retractors are not utilised to visualise surgical field	Low [1]	Low [1]	1	Remember to insert retractors to increase field of view
9.3	Palmar fascia not sufficiently divided to expose underlying structures	Low [1]	Medium [2]	2	Ensure palmar fascia is adequately divided to expose underlying structure
9.4	Soft tissue not retracted	Medium [2]	Low [1]	2	Remember to retract any soft tissue that is obscuring view
9.5	PB not sufficiently divided to expose underlying structures	Medium [2]	Medium [2]	4	Diathermy PB in a slow and controlled manner
9.6	TCL not sufficiently divided to expose underlying median nerve	Medium [2]	Medium [2]	4	Ensure to cut TCL centrally. Ensure median nerve is identified
9.7	TCL not release distally enough to level of fat pad	Medium [2]	Medium [2]	4	Extend incision through TCL until fat pad is visualised distally
9.8	Retractors not positioned proximally	Low [1]	Low [1]	1	Remember to reposition retractors when moving proximally
9.9	Above and below TCL not sufficiently cleared proximally	Medium [2]	Medium [2]	4	Ensure adequate clearance above and below TCL proximally
9.10	TCL not release proximally enough	Medium [2]	Medium [2]	4	Use your finger to check the TCL is adequately released proximally
9.11	Integrity of median nerve not inspected	Low [1]	Low [1]	1	Remember to check the median nerve from proximal to distal
10	Release tourniquet and haemostasis				
10.1	Tourniquet not released	Low [1]	Medium [2]	2	Remember to release the tourniquet
10.2	Haemostasis not achieved with diathermy	Medium [2]	Medium [2]	4	Ensure adequate haemostasis is achieved before wound closure particularly in a coagulopathic patient
11	Skin closure				
11.1	Too few sutures to skin	Medium [2]	Medium [2]	4	Ensure appropriate amount and spacing of sutures.
12	Postoperative wound care				
12.1	Wound not washed following wound closure	Low [1]	Low [1]	1	Remember to wash and dry the wound with sterile swabs
12.2	Not enough dressings applied to wound	Medium [2]	Medium [2]	4	Ensure nonadherent dressing is applied to the skin. Ensure the dressing is secure
12.3	Tourniquet not removed	Low [1]	Low [1]	1	Remember to remove tourniquet
13	WHO sign-out				
13.1	WHO sign-out checklist not completed	High [3]	Low [1]	3	Have checklist readily available in theatre

LA = local anaesthetic; SHERPA = systematic human error reduction and prediction approach; WHO = World Health Organization

Three subtasks were identified as high criticality, i.e. having the potential to cause permanent clinical effect. These included failure to expel air from the syringe before LA administration, failure to aspirate the syringe before infiltrating the local anaesthesia and failure to complete the WHO time-out checklist before skin incision. Although more subtasks identified errors that were likely to occur, those subtasks were deemed unlikely to have a noticeable clinical effect.

Each error was then classified into a behaviour type: action, retrieval, checking, selection or information communication. Of the four errors deemed to have a high occurrence probability, two were classified as action behaviours — one as a selection behaviour and one as a retrieval behaviour. The errors classified as action behaviours included failure to sterilise the skin before administration of LA and failure to infiltrate LA in a proximal-to-distal direction. The error classified as selection behaviour was selecting incorrect tourniquet size. Finally, the error with high probability of occurring that was classified as retrieval behaviour was failure to complete the World Health Organization (WHO) surgical sign-out. Errors classified as retrieval behaviours relate to information retrieval. Of the three errors that were deemed high criticality, two were classified as action behaviours and the third was classified as an information communication behaviour. The errors classified as action behaviours included failure to expel air from the syringe before LA administration, failure to aspirate the syringe before infiltrating the local anaesthesia. The error classified as an information communication behaviour was failure to complete the WHO time-out checklist before knife to skin.

## Discussion

When acquiring new skills during training, young surgeons are naturally more prone to error. It has been suggested that standardisation, using HRA, is an approach to reducing error during surgery. This study examined a commonly performed procedure and highlighted several important steps in open carpal tunnel decompression that were felt to have the potential for serious adverse outcome. Our study also provided a framework that can be utilised in surgical education and simulation-based training with the potential to aid in skill acquisition and error-reduction.

As described in other HRA reports, this study was led by healthcare clinicians rather than healthcare safety experts. In some industries, this type of analysis is carried out by a dedicated safety expert, such as the nuclear power industry. In this setting, however, it is more beneficial that the analysis is carried out by those performing the procedure to increase the validity of the analysis.^6^

An optimum technique for each procedure is necessary for standardisation to be effective. Standardisation was achieved in the current study, through the iterative process of HTA. It is difficult to apply HRA to emergency procedures or complex, major cases where surgical variation can be high and patient factors can play a large role. While aiming to reduce error and simplify processes, applying HRA to emergency cases may add further burden to an already time-sensitive procedure. More complex cases with a large degree of variability, can be more difficult to analyse. The optimum technique to carry out a procedure is therefore more applicable to procedures such as carpal tunnel release.

We identified failure to aspirate syringe during local anaesthetic as an error of potential high criticality. The true incidence of air embolism following intravascular injection of local anaesthesia in peripheral nerve surgery is unknown. However, the potential side effects are well reported and include ischemia, infarction and stroke.^25^ Accidental intravascular injection of LA is more widely documented and can result in a number of local and systemic complications such as inadequate anaesthesia, shooting pain, skin pallor or ischemia, and/or heart palpitations.^26^

A limitation of this study, seen throughout HRA methodologies both in healthcare and other industries, is the limited number of SMEs from a single institution available to provide input on constructing the HTA and developing the SHERPA analysis.^6,7^ This study included input from five SMEs from a single institution. The HTA was derived from a combination of SME input and literature review. However, input from further SMEs from other surgical specialties who also perform open carpal tunnel decompression such as orthopaedic surgery, may have benefited the SHERPA analysis.

While this generic framework for carrying out open carpal tunnel decompression could be applied to a number of other commonly performed procedures and utilised in surgical education and simulation, it must be noted that there will inevitably be some variation in clinical practice both across surgical specialities and across different surgical units. If HTA is to be used in teaching surgical trainees operating in surgical units across the globe, variation in clinical practice should allow for experts across specialities and across units to refine the HTA according to their own variation in technique for performing the individual procedures. However, if HTA is to be effective in reducing error, it is important that internal agreement be reached at each unit to prevent large variations in practice being taught to surgical trainees.

HRA has substantial implications for improvement in both the training and assessment of trainee surgeons. A standardised protocol that assigns risk to subtasks can guide trainees to improve their operative skills and can address concerns in allowing trainees to perform technically challenging steps, while enhancing patient safety by reducing errors.
